# Association between oral ulcerative lesions, oral infections, and fever during the pre-engraftment phase after hematopoietic cell transplantation: results from the Orastem study

**DOI:** 10.1007/s00520-026-11267-9

**Published:** 2026-10-02

**Authors:** A. M. G. A. Laheij, J. E. Raber-Durlacher, B. Hasséus, M. C. Huysmans, S. J. M. van Leeuwen, K. Garming Legert, J. E. Johansson, S. Isom, D. M. Kline, M. D. Hazenberg, F. R. Rozema, M. T. Brennan, I. von Bültzingslöwen, N. M. A. Blijlevens

**Affiliations:** 1 Department of Oral Medicine, Academic Centre for Dentistry Amsterdam (ACTA), University of Amsterdam and VU Amsterdam, Gustav Mahlerlaan 3004, Amsterdam, 1081 LA The Netherlands; 2 Department of Oral Maxillofacial Surgery, Amsterdam UMC location Vrije Universiteit Amsterdam, Amsterdam, The Netherlands; 3 Department of Oral medicine and Pathology, Institute of Odontology, The Sahlgrenska Academy, University of Gothenburg, Gothenburg, Sweden; 4 Department of Dentistry, Radboud University Medical Center, Nijmegen, The Netherlands; 5 Department of Dental Medicine, Karolinska Institutet, Huddinge, Sweden; 6 Department of Hematology and Coagulation, The Sahlgrenska Academy, University of Gothenburg, Gothenburg, Sweden; 7 Department of Biostatistics and Data Science, Wake Forest University School of Medicine, Winston Salem, NC USA; 8 Department of Hematology, Amsterdam UMC location University of Amsterdam, Amsterdam, The Netherlands; 9 Department of Oral Medicine/Oral and Maxillofacial Surgery, Wake Forest University School of Medicine, Charlotte, NC USA; 10 Department of Oral Microbiology and Immunology, Institute of Odontology, The Sahlgrenska Academy, University of Gothenburg, Gothenburg, Sweden; 11 Department of Hematology, Radboud University Medical Center, Radboud Institute for Medical Innovation, Nijmegen, The Netherlands

**Keywords:** Oral condition, Oral infection, Fever, Hematopoietic cell transplantation, Oral mucositis

## Abstract

**Purpose:**

The percentage of cases with fever of unknown origin after hematopoietic cell transplantation (HCT) is high. An often overlooked clinical infection in HCT recipients may be chronic inflammation from the oral cavity. Therefore, the aim of the present study was to assess fever in the pre-engraftment phase after HCT and to examine the relationship between oral foci of infection before and in the first weeks after HCT and fever.

**Methods:**

Adult patients, scheduled for autologous or allogeneic HCT, were included in this prospective, observational multicenter cohort study. Oral health was determined pre-HCT. Following HCT conditioning, fever was registered daily; and oral mucositis and other oral side effects were registered three times weekly.

**Results:**

Data from 186 patients were available. The mean age was 53.0 years (± 12.9), and 55.4% were male. The most common malignancies were multiple myeloma, acute myeloid leukemia, and lymphoma. In total, 69.4% of the patients developed fever and 43.1% had an oral ulcerative lesion. After correction for age, site, conditioning regimen, diarrhea, vomiting, and duration of leukopenia, oral ulceration size remained significantly associated with fever, especially when fever was defined as ≥ 38.5°C. Pre-HCT chronic dental and oral conditions showed no association with post-HCT fever.

**Conclusion:**

Fever was common during the pre-engraftment phase after HCT and significantly associated with the extent of oral ulcerations. In contrast, pre-HCT dental and oral conditions showed no association with post-HCT fever, highlighting the specific role of mucosal injury in febrile episodes.

## Introduction

Fever after hematopoietic cell transplantation (HCT) is a very frequent side effect that may herald life threatening complications, depending on the treatment regime, cause of fever, and patient-related variables [1, 2]. Allogeneic HCT is characterized by profound and prolonged immunosuppression and high rates of early cytokine-mediated fever and infectious complications [3]. Autologous HCT recipients generally experience shorter neutropenia, lower rates of severe infection, and a substantial contribution of noninfectious febrile events such as engraftment syndrome [4, 5]. Risk factors for fever and blood stream infections (BSI) include severe neutropenia, myeloablative conditioning regimens for HCT, use of certain immune and/or targeted treatments, mucositis grade 3–4 (WHO scale) and significant medical comorbidity, such as uncontrolled or progressive cancer, pneumonia, or other complex infections at presentation [2, 6, 7]. While neutropenia is traditionally considered to be the most important risk factor, recent studies point towards mucositis as being actually the most important risk factor for fever during neutropenia and BSI [4, 8, 9].

Documented BSI occur only in 10–15% of patients with hematological malignancies despite a stringent regimen of blood culture surveillance and empirical antibiotics. A clinical infection without a microbiologically confirmed pathogen occurs in 20–30%, mostly infections of the lower respiratory tract, gastrointestinal tract or the skin [10]. Microbiologically identified infections occur in 20–25% of episodes of febrile neutropenia [10, 11]. In 40–45% of episodes of febrile neutropenia, no clinical and/or microbial cause is identified [11]. Noninfectious causes of fever include progressive malignancy, blood transfusions, cytokine release syndrome, engraftment syndrome, idiopathic pneumonia syndrome, and drugs effects [1].

So, the percentage of cases with fever of unknown origin is high [11]. An often overlooked clinical infection in HCT recipients may be chronic, dental plaque induced, inflammation from the oral cavity such as periodontitis, periapical infections, and pericoronitis [12]. HCT-related oral complications such as oral mucositis or oral fungal and viral infections may also cause fever [4]. In immunocompromised patients, the hosts defense mechanisms are hampered and the symbiotic state of the host–microbiota interaction becomes dysbiotic [13, 14]. Opportunistic and pathogenic microorganisms may grow out and translocate through the ulcerated oral mucosa to the blood and cause a bloodstream infection, fever, or even sepsis. Yet, oral foci rarely exacerbate or are rarely associated with bloodstream infections after cytotoxic chemotherapy [15–17]. However, studies are difficult to compare as outcome measures differ and fever is rarely used as an outcome measurement. Therefore, the aim of the present study was to assess fever in the pre-engraftment phase after HCT and to examine the relationship between oral foci of infection and inflammation before and in the first weeks after HCT, and fever.

## Materials and methods

This study is reported in accordance with the STROBE statement for observational cohort studies.

### Study design and selection

This was a prospective, multicenter, observational study of adult patients (≥ 18 years old) scheduled for autologous or allogeneic HCT and included in the Orastem study [18], clinical trial number: not applicable. For the purpose of this study, patients were recruited at intervals between 2011 and 2018 at the following sites: Sahlgrenska University Hospital, Gothenburg and Karolinska University Hospital Huddinge, Stockholm, Sweden; Atrium Health Carolinas Medical Center, Charlotte, NC, USA; Amsterdam UMC, University of Amsterdam, Amsterdam, The Netherlands and Radboud University Medical Center, Nijmegen, The Netherlands. Data from BC Cancer, Vancouver, Canada, were not complete and therefore not included in this sub analysis. The complete study protocol has been documented elsewhere [18]. Data on other sub-studies are published previously [19, 20].

### Medical ethical review procedure

The study received approval from the Medical Ethical Committees at each participating center (Netherlands: Amsterdam University Medical Center location AMC (NL52117.018.15), Sweden: Regional Ethical Review Board in Gothenburg (513–10, T939-16); USA: Wake Forest School of Medicine Institutional Review Board (IRB00080071). All participants provided written informed consent, and the study was performed in line with the principles of the Declaration of Helsinki, the Good Clinical Practice (GCP) guidelines and the World Medical Association.

### Assessments

All investigators were calibrated before the start of the study. Before HCT: oral health was assessed 1–8 weeks prior to the start of HCT through a comprehensive oral examination, combined with oral radiographs, and questions about oral complaints. The baseline oral assessment included the presence of pericoronitis, (partially) impacted teeth and roots, percentage plaque visible with the naked eye, the presence of subgingival calculus, the number of teeth with periodontal probing depths > 5 mm (assessed at six sites per tooth), percentage bleeding on probing, presence of periapical lesions, number of caries lesions into the dentin or pulp, oral hygiene habits, presence of mucosal condition, and oral symptoms. Urgent dental treatments were performed. Preventive protocols for oral mucositis consisted of oral cryotherapy during conditioning therapy in autologous HCT recipients and institutional oral care protocols.

After HCT: during hospitalization (the pre-engraftment phase), patients were visited bedside three times a week. They received an oral examination performed by calibrated staff and answered questionnaires about oral complaints. The presence and severity of oral mucositis was assessed by trained and calibrated examiners, three times weekly using the World Health Organization (WHO) toxicity scale [21] and the modified Oral Mucositis Assessment Scale (OMAS) [22]. The OMAS scale includes scores for redness (scores 0 = none, 1 = mild, 2 = severe) and ulcerations, in this study modified as total ulcerative area (0 = none, 1 = cumulative size total area < 1 cm^2^, 2 = cumulative size total area 1–3 cm^2^, 3 =  > 3 cm^2^) on nine different oral sites.

The Oral Mucositis Daily Questionnaire (OMDQ) was used to assess the impact of oral mucositis on pain and daily functioning, including mouth and throat soreness; limitation in swallowing, drinking, eating, talking, and sleeping from mouth and throat soreness; and severity of diarrhea. The items were scored on a 5-point Likert scale or a numerical rating scale ranging from 0 to 10 [23]. Other side effects were measured using the Common Terminology Criteria for Adverse Events (CTCAE v3.0) including vomiting and dry mouth. For all side effects, the maximum score per patient was used for analysis. The presence of an oral mucosal infection was recorded. A viral infection was based on the clinical presentation and symptoms of suspicious lesions and was confirmed by virological tests. A fungal infection was clinically diagnosed based on the presentation and symptoms only.

Body temperature was recorded daily tympanically, with the highest recorded temperature per day considered for analysis. Fever was defined as at least a temperature of ≥ 38.0°C. Local practice differed when blood cultures were taken; in Gothenburg and Charlotte, in case, patients had a body temperature of ≥ 38.0°C and in Nijmegen and Amsterdam when a patient had a temperature of ≥ 38.5°C. Standard procedure for blood cultures included an aerobic and anaerobic blood culture for at least 48 h in all sites. In Charlotte, all HCT patients had a tunneled catheter. In Gothenburg, patients had a non-tunneled double-lumen (autoHCT) or triple-lumen (alloHCT) central venous catheter. In Nijmegen and Amsterdam, the patients receiving a myeloablative conditioning regimen had a non-tunneled central venous catheter, while reduced intensity (RIC)/nonmyeloablative (NMA) patients did not. All patients receiving myeloablative conditioning received antibiotic, antiviral, and antifungal prophylaxis at the start of conditioning for HCT. Prophylactic protocols varied between study centers, with each center applying its own established procedures based on prior experience and the medical team's clinical decision. This variability reflects the diverse approaches to conditioning regimens designed to meet patient-specific needs and institutional practices. All patients received empirical antimicrobial therapy in case of fever during neutropenia.

Due to insufficient availability of neutrophil-specific data, the duration of neutropenia could not be directly assessed at all time points in this study. However, leukocyte count data were consistently available and deemed reliable and were therefore used as a surrogate marker for neutropenia. This approach is justified, as a leukocyte count below 0.5 × 10^9^/L typically corresponds to a similarly reduced neutrophil count. The duration of leukopenia (WBC < 0.5 × 10^9^/L) was calculated in days, serving as a proxy for the period of neutropenia.

### Data handling and statistical analysis

For each variable, the denominator represents the number of participants with available data for that specific variable. We performed available-case analyses and did not impute missing values. Missingness did not follow a consistent pattern across variables and could reflect incomplete documentation at a specific assessment time point, clinical condition of the patient, or logistical circumstances of data collection.

WHO oral mucositis were dichotomized into no ulcerative oral mucositis (grade 0 and 1) and ulcerative oral mucositis (grades 2, 3, and 4). Other oral foci, scores from the OMQD and CTCAE were collapsed, depending on the observed counts per category. Scores for any periodontal pockets > 5mm, caries lesions to dentin, pulp lesions, or periapical lesions left untreated positive assessment were recalculated and dichotomized as yes/no oral focus before HCT. Fever was dichotomized as yes/no per patient. Statistical analyses, both descriptive and inferential, were conducted using the SAS 9.4 (SAS Institute Inc, Cary, NC, USA). Since many patients were discharged after a little over 2 weeks, data were reported and used for analysis until day 17 after HCT, to avoid selection bias.

Fever was the dependent variable. As participating centers applied different temperature cutoffs for obtaining blood cultures, fever was defined as either ≥ 38.0°C or ≥ 38.5°C. Selection criteria for independent variables for the multivariate analysis were as follows: significant association in a simple multivariable analysis, occurrence with sufficient frequency and clinical relevance. Though some data were measured at multiple time points, fever was collapsed into a dichotomous variable (ever fever/never fever) and all other variables measured longitudinally were also collapsed with the worst observation being used for each participant. A simple multivariable logistic regression model adjusting for site and duration of leukopenia was used as an initial step of looking at associations of measures deemed clinically meaningful. This informed the measures to be included in the full multivariable logistic regression model. The full model contained site, age, conditioning regimen, OMAS mucositis score, diarrhea, vomiting grade, and duration of leukopenia. A *p*-value < 0.05 was considered statistically significant.

## Results

### Demographic characteristics

At one of the participating centers, patient questionnaires were not completed according to the study design, which led to insufficient data for analyses from that center (Vancouver, Canada). Further, 30 participants with insufficient data were excluded, meaning that out of the 239 participants in the Orastem study seen post-HCT, data from 186 patients were available for analyses in this study. The median age of all 186 participants was 56.0 years (range 18.0–76.0), and 103 (55.4%) were male. The most common malignancies were multiple myeloma (62, 33.3%), acute myeloid leukemia (44, 23.7%) and malignant lymphoma (26, 14.0%). Half of the patients underwent RIC/NMA conditioning regimen (95, 51.1%), see Table 1.

**Table 1:** Patient characteristics

	All	alloHCT	autoHCT
*N*	186	112	74
Age, median (range)	56.0 (18.0–76.0)	55.0 (18.0–76.0)	57.0 (30.0–69.0)
Gender			
Male	103 (55.4%)	62 (55.4%)	41 (55.4%)
Female	83 (44.6%)	50 (44.6%)	33 (44.6%)
Study site			
Nijmegen (RUNMC)	65 (34.9%)	41 (36.6%)	24 (32.4%)
Charlotte	53 (28.5%)	48 (42.9%)	5 (6.8%)
Amsterdam (AMC)	42 (22.6%)	14 (12.5%)	28 (37.8%)
Gothenburg	26 (14.0%)	9 (8.0%)	17 (23.0%)
Medical diagnosis			
Multiple myeloma	62 (33.3%)	1 (0.9%)	61 (82.4%)
Acute myeloid leukemia	44 (23.7%)	44 (39.3%)	0
Lymphoma	26 (14.0%)	17 (15.2%)	9 (12.2%)
Myelodysplastic syndrome	12 (6.5%)	12 (10.7%)	0
Acute lymphoid leukemia	11 (5.9%)	11 (9.8%)	0
Myelofibrosis	10 (5.4%)	10 (8.9%)	0
Chronic myelogenous leukemia	5 (2.7%)	5 (4.5%)	0
Chronic lymphocytic leukemia	4 (2.2%)	4 (3.6%)	0
Severe aplastic anemia	2 (1.1%)	2 (1.8%)	0
Other	10 (5.4%)	6 (5.4%)	4 (5.4%)
RIC/FIC^a^			
RIC/NMA^a^	95 (51.1%)	92 (82.1%)	3 (4.1%)
FIC/myeloablative^a^	91 (48.9%)	20 (17.9%)	71 (95.9%)
Conditioning regimen			
Melphalan	65 (34.9%)	2 (1.8%)	63 (85.1%)
Cyclophosphamide + TBI^b^	57 (30.6%)	57 (50.9%)	0
Busulfan ± cyclophosphamide	26 (14.0%)	25 (22.3%)	1 (1.4%)
Total body irradiation	19 (10.2%)	19 (17.0%)	0
BEAM	9 (4.8%)	0	9 (12.2%)
Cyclophosphamide	4 (2.2%)	3 (2.7%)	1 (1.4%)
Treosulfan	3 (1.6%)	3 (2.7%)	0
Other	3 (1.6%)	3 (2.7%)	0
GVHD prophylaxis			
Posttreatment cyclophosphamide	40 (21.5%)	40 (35.7%)	0
Methotrexate	19 (10.2%)	19 (17.0%)	0
Other	53 (28.5%)	53 (47.3%)	0
None—autologous	74 (39.8%)	0	74 (100%)

^a^Full intensity conditioning/reduced intensity conditioning/nonmyeloablative

^b^Total body irradiation

### Oral condition before HCT

The details of oral symptoms and manifestations of oral condition before HCT are described elsewhere [19]. In short, 17 patients (9.3%) had at least one tooth with pulpal exposure, 43 (23.2%) with asymptomatic apical periodontitis, 4 (2.2%) with symptomatic apical periodontitis, and 11 (6.0%) with partially impacted teeth. Then, 29 (16.7%) were left with a pocket >  5 mm left untreated, 74 (40.0%) with at least one caries lesion into dentin left untreated, and 24 (13.0%) with one or more periapical lesions left untreated. Then, 36 (19.4%) teeth were extracted, 6 (3.2%) teeth underwent root canal treatment, 33 (17.7%) teeth were restored, 27 (14.5%) patients underwent partial, and 31 (16.7%) full mouth subgingival scaling before HCT (Table 2).

**Table 2:** Dental condition prior to HCT, in number of teeth per patient; number of patients (*N*)

Dental condition		*N*(%)
Total number of teeth with caries into dentin or pulp at baseline examination	0	84 (45.4%)
	≥ 1	101 (54.6%)
Total number of teeth with pulpal exposure at baseline examination	0	166 (90.7%)
	≥ 1	17 (9.3%)
Total number of asymptomatic teeth with apical periodontitis at baseline examination	0	142 (76.8%)
	≥ 1	43 (23.2%)
Total number of symptomatic teeth with apical periodontitis at baseline examination	0	180 (97.8%)
	≥ 1	4 (2.2%)
Total number of partially impacted teeth that communicate with the oral cavity at baseline examination	0	171 (94.0%)
	≥ 1	11 (6.0%)
Total number of teeth with at least one deep pocket (> 5 mm) at baseline examination	0	100 (68.0%)
	≥ 1	47 (32.0%)
Total number of teeth with bleeding on probing at baseline examination	0	19 (4.4%)
	≥ 1	113 (85.6%)
Total number of teeth with subgingival calculus at baseline examination	0	120 (70.6%)
	≥ 1	50 (29.4%)
Number of teeth with a periodontal pocket > 5 mm left untreated	0	145 (83.3%)
	≥ 1	29 (16.7%)
Number of teeth with one or more carious lesions to dentin left untreated	0	111 (60.0%)
	≥ 1	74 (40.0%)
Number of teeth with periapical lesions left untreated	0	161 (87.0%)
	≥ 1	24 (13.0%)
Number of tooth extractions that have been performed after dental examination and before transplantation	0	150 (80.6%)
	≥ 1	36 (19.4%)
Number of teeth that had root canal therapy after dental examination and before transplantation	0	180 (96.8%)
	≥ 1	6 (3.2%)
Number of teeth that have been restored after dental examination and before transplantation	0	153 (82.3%)
	≥ 1	33 (17.7%)
Number of implants which have been removed after dental examination and before transplantation	0	185 (100%)
	≥ 1	0
Has subgingival scaling been performed after dental examination and before transplantation?	No	128 (68.8%)
	Yes-partial	27 (14.5%)
	Yes-full mouth	31 (16.7%)

### Oral conditions after HCT during pre-engraftment period

In the 186 patients reported in this study, 38.1% of the patients experienced ulcerative oral mucositis (WHO score ≥ 2) and 43.1% had an ulcerative/pseudomembrane lesion (OMAS) during hospitalization. Ulcerative oral mucositis peaked between days + 9 and 11. Twenty-eight patients (11.8%) developed an oral mucosal infection during hospitalization, of which most were fungal. More details about oral mucositis and oral infections during the pre-engraftment period will be described in a different manuscript (Skallsjö et al., submitted). Thirty-one oral conditions requiring intervention were identified during the pre-engraftment phase (Table 3).

**Table 3:** Objective dental findings during pre-engraftment phase

		Fever	
	All	No	Yes
Sharp tooth edge that requires attention			
No	179 (98.9%)	56 (100%)	123 (98.4%)
Yes	2 (1.1%)	0	2 (1.6%)
Purulence with dental infection			
No	180 (99.4%)	55 (98.2%)	125 (100%)
Yes	1 (0.6%)	1 (1.8%)	0
Deep dental caries causing symptoms			
No	181 (100%)	56 (100%)	125 (100%)
Yes	0	0	0
Swelling with dental infection			
No	180 (99.4%)	56 (100%)	124 (99.2%)
Yes	1 (0.6%)	0	1 (0.8%)
Pulpitis			
No	181 (100%)	56 (100%)	125 (100%)
Yes	0	0	0
Pericoronitis			
No	180 (99.4%)	56 (100%)	124 (99.2%)
Yes	1 (0.6%)	0	1 (0.8%)
Tooth fracture			
No	177 (97.8%)	54 (96.4%)	123 (98.4%)
Yes	4 (2.2%)	2 (3.6%)	2 (1.6%)
Filling fracture			
No	178 (98.3%)	55 (98.2%)	123 (98.4%)
Yes	3 (1.7%)	1 (1.8%)	2 (1.6%)
Loose tooth			
No	180 (99.4%)	56 (100%)	124 (99.2%)
Yes	1 (0.6%)	0	1 (0.8%)
Cracked tooth			
No	181 (100%)	56 (100%)	125 (100%)
Yes	0	0	0
Other			
No	163 (90.1%)	51 (91.1%)	112 (89.6%)
Yes	18 (9.9%)	5 (8.9%)	13 (10.4%)

### Fever during pre-engraftment period

In total, 69.4% (*n* = 129) of the patients developed fever (≥ 38.0°C) in the pre-engraftment period after HCT. In case fever was defined as ≥ 38.5°C, 55.9% (*n* = 104) developed fever. According to the institutional protocol, blood cultures were taken from 104 patients. The blood culture was positive in 50/104 (48%) patients. The identified microbiological species were very diverse. Of the 50 positive cultures, most were identified as *Staphylococcus epidermidis* (12/50), *Escherichia coli* (5/50), and *Staphylococcus aureus* (4/50), and 12 of out 50 were polymicrobial. This means that 48% of the fever episodes were microbiologically identified infections. Data on the presumed clinical cause of infection in our patients were not available.

### Associations with fever

The presence of any oral pathologies (periodontal pockets >  5 mm, carious lesions to dentin, pulp lesions, or periapical lesions left untreated before HCT) was not significantly related to fever (OR 1.09; CI 0.52–2.28; *p* = 0.8218). However, baseline variables age (5-year increase OR 0.84; CI 0.72–0.97; *p* = 0.0218), site (*p* = 0.004), and conditioning regimen (full-intensity conditioning/myeloablative vs. RIC/NMA; OR 2.40; CI 1.07–5.39; *p* = 0.0357) were significantly associated with fever. The distribution of patient reported assessments during the pre-engraftment phase and fever are depicted in Table 4. Ulcerative oral mucositis, duration of leukopenia, vomiting, diarrhea, erythema, and mouth and throat soreness were also significantly associated with fever (Table 4).

**Table 4:** Distribution of oral health assessments and simple univariable analyses with the presence of fever during pre-engraftment phase after HCT

Assessment	No fever ever (*N* (%))	Fever ever (*N* (%))	Odds ratio (CI)	*p*-value
*Oral mucositis (WHO)*				
Grade 0–1	39 (34.8%)	73 (65.2%)	Reference	0.0621
Grade 2–4	17 (24.6%)	52 (75.4%)	2.04 (0.96, 4.30)	
*Oral ulceration/pseudomembrane (modified OMAS)*				
No lesion/normal	35 (34.0%)	68 (66.0%)	Reference	0.001*
< 1 cm^2^ total area	17 (45.9%)	20 (54.1%)	0.59 (0.25, 1.40)	
≥ 1 cm^2^ total area	4 (9.8%)	37 (90.2%)	6.81 (2.10, 22.09)	
*Oral erythema (OMAS)*				
Normal	32 (38.6%)	51 (61.4%)	Reference	0.0116*
Not severe/severe	23 (24.0%)	73 (76.0%)	2.65 (1.24, 5.64)	
*Mouth and throat soreness (during the last 24 h)*				
No soreness	26 (41.3%)	37 (58.7%)	Reference	0.0239*
A little soreness	14 (32.6%)	29 (67.4%)	0.82 (0.31, 2.21)	
Moderate soreness	8 (26.7%)	22 (73.3%)	1.16 (0.38, 3.51)	
Quite a lot/extreme soreness	8 (17.8%)	37 (82.2%)	4.05 (1.47, 11.14)	
*Diarrhea (during the last 24 h)*				
No diarrhea	17 (51.5%)	16 (48.5%)	Reference	0.0060*
A little diarrhea	9 (39.1%)	14 (60.9%)	1.48 (0.44, 5.05)	
Moderate diarrhea	15 (35.7%)	27 (64.3%)	1.62 (0.58, 4.49)	
Quite a lot/severe diarrhea	15 (18.1%)	68 (81.9%)	5.02 (1.90, 13.26)	
*Dry mouth*				
Grade 0	11 (55.0%)	9 (45.0%)	Reference	0.0725
Grade 1	29 (31.2%)	64 (68.8%)	2.49 (0.87, 7.18)	
Grade 2–3	16 (23.5%)	52 (76.5%)	3.77 (1.21, 11.74)	
*Vomiting*				
Grade 0	28 (39.4%)	43 (60.6%)	Reference	0.0036*
Grade 1	15 (35.7%)	27 (64.3%)	1.24 (0.50, 3.04)	
Grade 2–3	13 (19.1%)	55 (80.9%)	4.56 (1.83, 11.37)	
*Duration of leukopenia (median, 25th perc;75th perc)*	7.0 (2.0–10.0)	9.0 (6.0–12.0)	1.10 (1.02, 1.18)	0.0144*

*Marks significant association

Odds ratios and *p*-value are from a multivariable model looking at presence of fever and adjust for duration of leukopenia (days white blood count < 0.5) and clinical site

In the full multivariate model analyses, age, study site, conditioning regimen, size of the oral ulceration, duration of leukopenia in days, diarrhea, and vomiting (as surrogate markers for gastrointestinal mucositis) were entered as independent variables (Table 5). Larger oral ulceration size (≥ 1 cm^2^) and vomiting more often were significantly associated with the presence of fever in the pre-engraftment phase after HCT, after adjusting for the other variables, with an odds ratio of 6.36 and 3.72, respectively. Duration of leukopenia was also significantly associated with fever, with an odds ratio of 1.09.

**Table 5:** Results of multivariate analysis with fever (≥ 38.0°C) as dependent variable

Effect	OR	95% Wald CI		*p*-value
*Oral ulceration/pseudomembrane (OMAS)*				
No lesion/normal	Reference			0.0028*
< 1 cm^2^ total area	0.54	0.21	1.40	
≥ 1 cm^2^ total area	6.36	1.75	23.11	
*Diarrhea (during the last 24 h)*				
No diarrhea	Reference			0.0904
A little diarrhea	1.51	0.35	6.49	
Moderate diarrhea	1.81	0.54	6.03	
Quite a lot/severe diarrhea	4.27	1.28	14.29	
*Vomiting*				
Grade 0: none	Reference			0.0251*
Grade 1: 1 episode in 24 h	1.03	0.36	2.93	
Grade 2/3: ≥ 2 episodes in 24 h	3.72	1.35	10.27	
*Duration of leukopenia*	1.09	1.00	1.19	0.0452*

*Marks significant association

In addition to the variables included in Table 5, the multivariable model also adjusts for age, conditioning regimen, and site.

When fever was cut off at a temperature of ≥ 38.5°C instead of ≥ 38.0°C, the result of the multivariate analysis was slightly different. Only the size of the oral ulceration remained significantly associated with fever, while vomiting, diarrhea, and duration of leukopenia were not (Table 6). Oral ulceration size and diarrhea were not significantly associated with each other (*p* = 0.1022).

**Table 6:** Results of multivariate analysis with fever (≥ 38.5°C) as dependent variable

Effect	OR	95% Wald CI		*p*-value
*Oral ulceration/pseudomembrane (OMAS)*				
No lesion/normal	Reference			0.0007*
< 1 cm^2^	0.68	0.29	1.62	
≥ 1 cm^2^	6.02	2.13	17.03	
*Diarrhea (during the last 24 h)*				
No diarrhea	Reference			0.4143
A little diarrhea	1.35	0.35	5.14	
Moderate diarrhea	1.31	0.43	4.01	
Quite a lot/severe diarrhea	2.26	0.77	6.61	
*Vomiting*				
Grade 0: none	Reference			0.0851
Grade 1: 1 episode in 24 h	1.16	0.45	2.97	
Grade 2/3: ≥ 2 episodes in 24 h	2.47	1.08	5.65	
*Duration of leukopenia*	1.05	0.97	1.13	0.2263

*Marks significant association

In addition to the variables included in Table 6, the multivariable model also adjusts for age, conditioning regimen, and site.

## Discussion

The aim of the present study was to assess fever in the pre-engraftment phase after HCT and to examine the relationship between fever and oral foci of infection. Fever was common during the pre-engraftment phase and there was a significant and relevant association between the total size of oral ulcerations and fever. Pre-HCT chronic dental and oral conditions (such as caries into the dentin or pulp, periapical lesions, periodontal inflammation, pocket depth, and partially impacted teeth) were not associated with the presence of fever after HCT. Exacerbations of chronic dental and periodontal condition during the engraftment phase were rare.

The duration of neutropenia is traditionally considered to be the most significant risk factor for fever, but our analysis shows a more nuanced pattern. Larger oral ulcerations showed the most consistent association with fever across the two fever thresholds in the fitted models. A recent study reported about the relationship between fever, septicemia, and (oral and gastrointestinal) mucositis in as many as 71,780 hospitalized leukemia patients in the USA [24]. Oral ulcerative mucositis and gastrointestinal mucositis were significantly associated with fever during neutropenia, but not with septicemia. This association was present in patients receiving chemotherapy and high-dosed conditioning regimens preparing for HCT [24]. Others reported about the association between intestinal mucositis and blood stream infections irrespective of duration of neutropenia [8, 9, 25].

In neutropenic patients, the threshold used to define fever can significantly influence both early detection of infection and the initiation of empiric antimicrobial therapy. In patients with neutropenia due to chemotherapy, fever is defined by both the Infectious Disease Society of America and the National Comprehensive Cancer Network as a single oral temperature measurement greater than or equal to 38.3°C or greater than 38.0°C sustained over at least 1 h [26]. Some centers use the higher cutoff of ≥ 38.5 °C to reduce the number of false positives and potentially avoid unnecessary broad-spectrum antibiotic use. The association between oral mucositis and fever in our study was stronger when fever was defined as ≥ 38.5 °C. This suggests that higher fever thresholds may better reflect clinically significant inflammation or secondary infection related to mucosal barrier injury. Ulcerative oral mucositis facilitates microbial translocation and related-cytokine release [27], which may drive a more pronounced febrile response. Using a higher fever cutoff may therefore capture the more clinically relevant febrile mucositis [28].

Oral foci of infection pose a potential risk to HCT patients, particularly during the neutropenic phase, which typically occurs one to 2 weeks post-transplantation. A key question is whether pre-HCT oral foci contribute significantly to infectious complications during this vulnerable neutropenic period. In the present study, chronic oral foci and dental condition assessed pre-HCT, including caries into the dentin or pulp, periapical lesions, periodontal inflammation, and partially impacted teeth, were not associated with the occurrence of fever. Most of these oral foci and dental conditions were eliminated before HCT. This suggests that the risk of chronic oral infections leading to febrile episodes in the early engraftment period after HCT is low.

Historically, it has been recommended that periodontitis (chronic inflammatory disease) is treated prior to HCT to reduce the risk of infectious complications [29, 30]. However, more recent studies have reported no significant association between preexisting oral foci of infection and post-HCT fever [12, 15, 17, 31, 32]. A large cohort study involving 500 HCT recipients found that the presence of three or more oral foci was associated with prolonged hospitalization but not with early post-transplant mortality [33]. In another cohort of 350 HCT patients, only two patients had odontogenic complications after HCT [16]. A recent retrospective study in 15,911 patients found that pretreatment oral management may reduce posttransplant infections but not severe oral mucositis and mortality [12]. These findings support a more nuanced approach to dental management of chronic oral infections prior to HCT, and more research into personalized management protocols is necessary.

Although no significant association between fever and oral mucositis was observed using the WHO classification for oral mucositis, a significant and meaningful relationship emerged when using the OMAS score. This discrepancy may be attributed to fundamental differences between the scoring systems. The OMAS score quantifies the size and extent of ulcerative lesions, offering a more detailed description of the lesions itself. In contrast, the WHO classification considers the functional impact of oral mucositis, such as the patient’s ability to eat. As a result, patients may present with large ulcerative lesions that do not interfere with oral intake, resulting in low WHO scores but high OMAS scores. The association between larger ulcerative lesions and fever may be explained by heightened local inflammation and increased risk of microbial translocation during neutropenia.

The proportion of microbiologically confirmed infections in our study population was 48%, which is notably higher than the average reported rates of 20–25% in similar cohorts [10, 11]. Because treatment centers in this study measured fever more frequently and obtained cultures more often, a higher number of positive blood cultures was observed. Unfortunately, our dataset did not include information on clinical infections originating from non-oral sites, such as the urinary tract, skin, or lungs. As a result, we were unable to determine the overall percentage of clinical infections or to compare the relative risk of infections at extra-oral sites with those associated with oral ulcerative mucositis. Future studies would benefit from a comprehensive assessment of all potential infection sites, integrated with microbiological findings, to better understand the full spectrum and distribution of infectious complications in HCT recipients.

Although oral mucositis is primarily induced by cancer treatment, both patient-related and treatment-related factors as well as supportive care interventions contribute to its risk [34]. For instance, oral cryotherapy and institutional oral care protocols, which were implemented as part of the treatment protocol in all participating centers in this study, have been shown to reduce the risk of oral mucositis and related fever in patients receiving high-dose melphalan preparative regimens for HCT [35]. Other interventions that have shown to reduce the risk of oral mucositis are photobiomodulation therapy (PBMT) and KGF-1 iv (in autologous HCT with a conditioning regimen that includes high-dose CT and TBI). GM-CSF and glutamine do not prevent oral mucositis [34].

Additionally, although oral dysbiosis has been associated with an increased risk of oral mucositis in previous studies [13, 14, 27], such data were not available for all patients in our study. Antibiotic use, which also disrupts the oral commensal flora, has not been shown to reduce the risk of oral mucositis [36]. Emerging microbial-based strategies such as applying prebiotics or microbial transplantations may offer promising avenues for reducing the incidence and severity of oral mucositis and warrant further investigation [27].

This study included data from multiple treatment centers, each with its own treatment protocols and observational practices, which may have contributed to variations in outcomes. We observed differences in the incidence of fever between the centers, which are likely attributable to variations in treatment protocols, such as surveillance strategies, antibiotic use, or other supportive care measures. Besides site, age was also significantly associated with fever. With every 5-year increase in age, the risk of fever decreased. Since age and site were not primary points of interest, we corrected for these variables in the multivariate analyses.

To conclude, fever was common during the pre-engraftment phase after HCT and was significantly associated with the extent of oral ulcerations. In contrast, pre-HCT dental and oral conditions showed no association with post-HCT fever, highlighting the specific role of mucosal injury in febrile episodes during this phase.

## Data Availability

Research data cannot be shared publicly because all data are stored at and belong to the University of Gothenburg (GU), and there are ethical and legal restrictions on sharing our data set according to Swedish law. Study data contain sensitive patient information that can be connected to individual patients. It may be possible to identify and connect personal information from the data set, even though data is de-identified. All relevant data within the paper are at group level.
